# Measurement of pre‐treatment inflammatory cytokine levels is valuable for prediction of treatment efficacy to tumor necrosis factor inhibitor in axial spondyloarthritis patients

**DOI:** 10.1111/1756-185X.14353

**Published:** 2022-06-12

**Authors:** Fei Peng, Fengyun Chen, Huijun Wen, Jie Bai, Yuping Tian

**Affiliations:** ^1^ Department of Traditional Chinese Medicine, Section of Western Medicine Foundation Teaching and Research Baoji Vocational & Technical College Baoji China; ^2^ Department of Hematology and Rheumatology Baoji Central Hospital Baoji China; ^3^ Department Neurology Baoji Central Hospital Baoji China; ^4^ Department of Rheumatology and Immunology No.215 Hospital of Shaanxi Nuclear Industry Xianyang China

**Keywords:** Assessment in SpondyloArthritis International Society 40 response, axial spondyloarthritis, interleukin‐17A, interleukin‐6, tumor necrosis factor inhibitor

## Abstract

**Aim:**

To evaluate the correlation of inflammatory cytokines with the treatment response to tumor necrosis factor inhibitor (TNFi) in axial spondyloarthritis (axSpA) patients.

**Methods:**

This study enrolled 86 axSpA patients and 20 healthy controls (HCs). Inflammatory cytokines including tumor necrosis factor‐α (TNF‐α), interleukin (IL)‐1β, IL‐6, IL‐12, IL‐17A, IL‐21, IL‐23, and IL‐32 were determined in serum samples of axSpA patients before treatment and in HCs after enrollment. All patients received 40 mg adalimumab every 2 weeks for 12 weeks; meanwhile, ASAS40 (40 criteria of the Assessment by the SpondyloArthritis International Society) response rates were evaluated at weeks 2, 4, 8, and 12.

**Results:**

Most inflammatory cytokines were elevated in axSpA patients compared with HCs (all *P* < 0.05) except for IL‐32 (*P* = 0.101). In axSpA patients, ASAS40 response rates were 0%, 19.5%, 34.5%, 47.1%, and 56.3% at weeks 0, 2, 4, 8, and 12, respectively. Baseline [interquartile range] IL‐6 (47.3 [32.5‐53.4] pg/mL vs 31.7 [23.0‐50.9] pg/mL, *P* = 0.005) and IL‐17A (127.9 [90.7‐149.5] pg/mL vs 96.6 [56.1‐112.6] pg/mL, *P* < 0.001) were higher in axSpA patients with ASAS40 response compared with those without ASAS40 response, while baseline TNF‐α, IL‐1β, IL‐12, IL‐21, IL‐23, and IL‐32 were not different between them (all *P* > 0.050). Multivariate logistic regression analysis disclosed that baseline IL‐17A (*P* = 0.037), C‐reactive protein (*P* = 0.012), and history of TNF inhibitor (*P* = 0.029) were independently associated with ASAS40 response. Furthermore, baseline IL‐17A, C‐reactive protein, history of TNFi, and their combination had an acceptable to good ability for predicting ASAS40 response.

**Conclusion:**

Measurement of pre‐treatment inflammatory cytokine levels is valuable for predicting treatment efficacy of TNFi in axSpA patients.

## 1 INTRODUCTION

Axial spondyloarthritis (axSpA), a chronic inflammatory disease leading to vertebral and sacroiliac fusion, is characterized by lower back pain and back stiffness in the morning.^1^, ^2^, ^3^ Even though there is still no cure for axSpA patients, the application of tumor necrosis factor‐α (TNF‐α) inhibitor in the treatment of axSpA ameliorates the disease status, including controlling the inflammation, decreasing the disease activity, and improving the health‐related quality of life.^4^, ^5^, ^6^ However, the annual cost of TNF inhibitor is high; in addition, a certain proportion of axSpA patients stop the TNF inhibitor therapy because of the lack of efficacy and the presence of adverse events.^7^, ^8^, ^9^ Hence, it is essential to find biomarkers to predict the TNF inhibitor response in axSpA patients.

Inflammatory cytokines, including TNF‐α, interleukin (IL)‐1β, IL‐6, IL‐12, IL‐17A, IL‐21, IL‐23, and IL‐32, are involved in regulating the immunoreaction.^10^, ^11^, ^12^, ^13^ For instance, IL‐1β may induce the inflammatory response and oxidative stress through the Rho‐kinase/IκB‐α/NF‐κB activation mechanism,^10^ and the activation of the IL‐23/IL‐17 axis may contribute to the inflammatory response by secretion of a range of pro‐inflammatory cytokines (including IL‐6, TNF‐α, and IL‐1). T‐cell‐attracting and neutrophil‐attracting chemokines including CCL2, CCL7, CXCL1, and CXCL2.^11^ Previous studies have reported that IL‐1β, IL‐17A, and TNF‐α may predict the treatment response to TNF inhibitors in inflammatory or rheumatic diseases, such as psoriasis and Crohn disease.^14^, ^15^ However, their relation to the TNF inhibitor response in axSpA patients is still obscure. Therefore, this study analyzed inflammatory cytokine levels in axSpA patients treated with TNF inhibitor and further aimed to investigate their correlation with TNF inhibitor treatment response in axSpA patients.

## 2 MATERIALS AND METHODS

### 2.1 Participants

After being approved by the Institutional Review Board, this study consecutively recruited 86 individuals with axSpA to receive adalimumab treatment between June 2018 and December 2019. The criteria for enrollment were set as follows: (a) confirmed diagnosis of axSpA following the ankylosing spondylitis classification criteria set by the SpondyloArthritis International Society;^16^ (b) axSpA activity meeting the criterion: Bath Ankylosing Spondylitis Disease Activity Index (BASDAI) greater than 4 (on a 0‐10 cm visual analog scale [VAS]) and Ankylosing Spondylitis Disease Activity Score based on C‐reactive protein (ASDAS‐CRP) greater than 2.1; (c) age older than 18 years; (d) no use of biologics in previous 3 months before recruitment; (e) willing to use adalimumab continuously for at least 12 weeks. The exclusion criteria were set as (a) contraindications to adalimumab (eg, infection, tuberculosis, active hepatitis B); (b) complicated with solid tumor or hematologic malignancies; (c) patients who were highly unable to benefit from adalimumab therapy (including patients who were known to have no response to previous adalimumab treatment or patients who were known to have no response to more than two kinds of other TNF inhibitors); (d) pregnant and lactating female patients. At the same time, another 20 age‐ (20‐40 years) and gender‐ (male to female as 4:1 ratio) matched healthy controls (HCs) were also enrolled. The exclusion criteria were also suitable for the HCs. Each participant signed informed consent.

### 2.2 Collection of baseline characteristics and samples

For axSpA patients only, each eligible axSpA patient received baseline (week 0) examinations and assessments. Clinical features such as demographics and disease history were documented through clinic inquiry. Subsequently, estimates of disease activity were carried out, including ASDAS‐CRP, BASDAI by VAS (0‐10 cm), Bath Ankylosing Spondylitis Functional Index (BASFI) by VAS (0‐10 cm), total back pain by VAS (0‐10 cm), and Patient's Global Assessment of Disease Activity (PGADA) by VAS (0‐10 cm). Meanwhile, blood was sampled from all patients for necessary biochemical tests and study determination.

### 2.3 Determination of inflammatory cytokines

A venous blood sample for study use was collected in a serum separator tube. Serum was isolated by centrifugal separation at 1000 ***g*** for 20 minutes, immediately used to determine inflammatory cytokines, including TNF‐α, IL‐1β, IL‐6, IL‐12, IL‐17A, IL‐21, IL‐23, and IL‐32. The determination of inflammatory cytokines was completed by enzyme‐linked immunosorbent assay (ELISA) using Human Quantikine ELISA Kits (R&D Systems, Minneapolis, MN, USA). The assay was carried out referring to the complete assay procedure of the kits. In brief, 100 μL of assay diluent and 50 μL of standards, control, or sample were added to each well and incubated for 2 hours. After that, each well was washed four times, and 100 μL of the conjugate was added to each well, followed by incubation for 1 hour and cleaning four times. Subsequently, 200 μL of substrate solution was added to each well, followed by incubation at room temperature for 30 minutes avoiding light. Finally, 50 μL of stop solution was added to each well, followed by immediately reading absorbance at 450 nm. The standard curve was fitted, which was used for calculating the concentration of unknown samples.

### 2.4 Administration of adalimumab and evaluation

AxSpA patients in this study received a subcutaneous injection of 40 mg adalimumab (Abbott Laboratories, Chicago, IL, USA) every 2 weeks for 12 weeks. Evaluation of treatment response was started at week 2 after initiation of adalimumab treatment, which was then performed at weeks 4, 8, and 12, respectively. The Assessment by SpondyloArthritis International Society 40 improvement criteria (ASAS40) were adopted to evaluate treatment response. ASAS40 response was defined as the improvement of at least 40% and at least 2 units in at least three domains on a scale of 10, without worsening at all in the remaining domain, where the territories comprised global patient assessment, pain, function, and inflammation (mean of BASDAI questions 5 and 6).^16^ In the analysis, patients who achieved an ASAS40 response at week 12 were classified as response patients, and the others without achievement of an ASAS40 response at week 12 were classified as non‐response patients.

### 2.5 Statistical analysis

The statistical analysis process and graph plotting were completed using SPSS 24.0 (IBM Corp., Armonk, NY, USA) and graphpad prism 6.01 software (GraphPad Software Inc., San Diego, CA, USA). The Kolmogorov‐Smirnov test examined the normality of the continuous variable. The distribution of inflammatory cytokines was illustrated using a box plot, and the Wilcoxon rank sum test determined the comparison of inflammatory cytokines. Logistic regression analysis for ASAS40 response at week 12 was performed. The forward stepwise methodology was applied to screen the independent prediction factors, further estimated for performance by receiver operating characteristic (ROC) curve analysis. The optimal specificity and sensitivity in the ROC curve analysis were calculated at the best cut‐off point, which was screened out from the values that gave the maximum specificity and sensitivity. A *P* value less than 0.05 indicated a statistical significance.

## 3 RESULTS

### Baseline characteristics of axSpA patients

The mean age of axSpA patients was 33.1 ± 8.7 years; 13 (15.1%) were female and 73 (84.9%) were male. The disease duration was 6.0 (interquartile range [IQR] 3.5‐9.0) years. Twenty‐three (26.7%) patients had a history of TNF inhibitor treatment, but the other 63 (73.3%) patients did not. No patients were on conventional synthetic disease‐modifying antirheumatic drugs currently and none had a history of use of IL‐17A inhibitors. Additionally, there were 77 (89.5%) patients who were HLA‐B27‐positive and 9 (10.5%) were HLA‐B27‐negative. The baseline CRP and erythrocyte sedimentation rate levels were 27.8 (IQR 18.7‐40.6) mg/L and 30.3 (IQR 20.5‐47.1) mm/h, respectively. BASDAI scores and ASDAS‐CRP were 6.2 (IQR 5.5‐6.8) and 4.0 (IQR 3.3‐4.6), respectively. Other baseline characteristics of axSpA patients, including BASFI score, total back pain score, and PGADA score, are listed in Table 1.

**TABLE 1:** Baseline characteristics of axSpA patients

Items	axSpA patients (N = 86)
Age (years), mean ± SD	33.1 ± 8.7
Gender, n (%)	
Male	73 (84.9)
Female	13 (15.1)
Disease duration (years), median (IQR)	6.0 (3.5‐9.0)
History of TNF inhibitor, n (%)	
Yes	23 (26.7)
No	63 (73.3)
HLA‐B27 positive, n (%)	
Yes	77 (89.5)
No	9 (10.5)
CRP (mg/L), median (IQR)	27.8 (18.7‐40.6)
ESR (mm/H), median (IQR)	30.3 (20.5‐47.1)
BASDAI score, median (IQR)	6.2 (5.5‐6.8)
BASFI score, median (IQR)	4.7 (4.0‐5.6)
Total back pain score, median (IQR)	7.0 (5.8‐7.3)
PGADA score, median (IQR)	7.0 (6.0‐7.0)
ASDAS‐CRP, median (IQR)	4.0 (3.3‐4.6)

Abbreviations: ASDAS‐CRP, Ankylosing Spondylitis Disease Activity Score with C‐reactive protein; axSpA, axial spondyloarthritis; BASDAI, Bath Ankylosing Spondylitis Disease Activity Index; BASFI, Bath Ankylosing Spondylitis Functional Index; CRP, C‐reactive protein; ESR, erythrocyte sedimentation rate; HLA‐B27, human leukocyte antigen B27; IQR, interquartile range; PGADA, Patient Global Assessment of Disease Activity; SD, standard deviation; TNF, tumor necrosis factor.

### Baseline levels of inflammatory cytokines in axSpA patients and HCs

In axSpA patients, the baseline levels of TNF‐α, IL‐1β, IL‐6, IL‐12, IL‐17A, IL‐21, IL‐23, and IL‐32 were 55.3 (IQR 42.7‐71.3) pg/mL, 6.1 (4.2‐8.6) pg/mL, 41.7 (29.2‐52.5) pg/mL, 62.9 (50.9‐89.5) pg/mL, 108.8 (76.6‐133.4) pg/mL, 248.7 (166.5‐378.7) pg/mL, 79.7 (49.7‐98.1) pg/mL, and 37.9 (25.2‐65.1) pg/mL, respectively. In HCs, the baseline inflammatory cytokines were as follows: 38.8 (IQR 30.3‐54.4) pg/mL for TNF‐α, 2.6 (1.8‐4.4) pg/mL for IL‐1β, 23.5 (17.1‐44.0) pg/mL for IL‐6, 48.5 (27.9‐70.8) pg/mL for IL‐12, 57.0 (43.4‐79.5) pg/mL for IL‐17A, 143.0 (120.6‐212.5) pg/mL for IL‐21, 48.6 (35.7‐70.8) pg/mL for IL‐23, and 31.4 (17.5‐48.5) pg/mL for IL‐32 (Figure S1). By comparison, most baseline inflammatory cytokines were elevated in axSpA patients compared with HCs (all *P* < 0.05) except for IL‐32 (*P* = 0.101).

### ASAS40 response rate to adalimumab in axSpA patients

**FIGURE 1:**
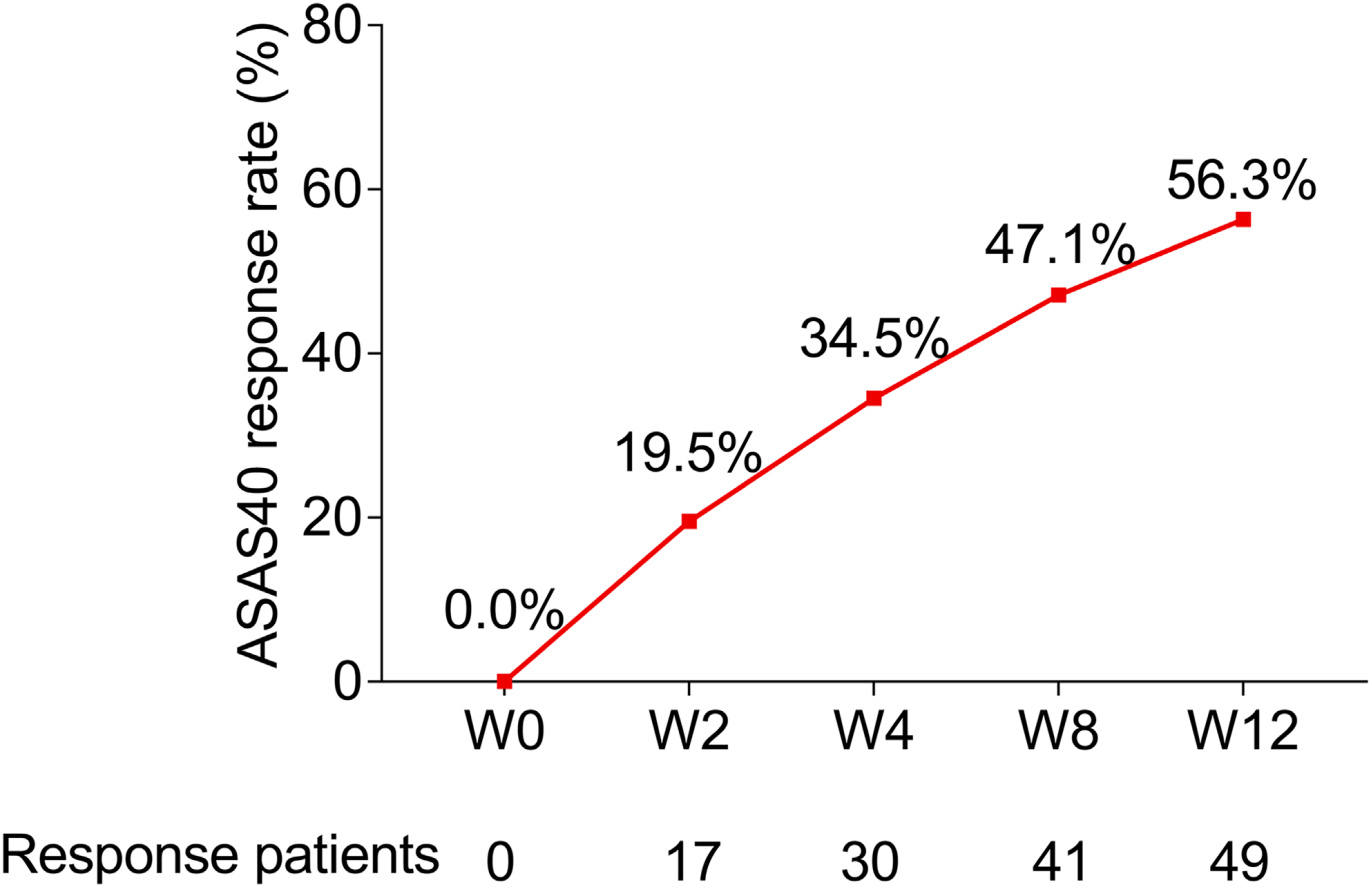
ASAS40 response rate after treatment with adalimumab; ASAS40, Assessment in SpondyloArthritis international Society 40; W0, week 0; W2, week 2; W4, week 4, W8, week 8; W12, week 12

Patients were continually followed up for a full 12 weeks and ASAS40 response rates were evaluated at several time points, which disclosed that 0%, 19.5%, 34.5%, 47.1%, and 56.3% axSpA patients achieved ASAS40 response at weeks 0, 2, 4, 8, and 12, respectively (Figure 1).

### Correlation of baseline inflammatory cytokine levels with ASAS40 response in axSpA patients

**FIGURE 2:**
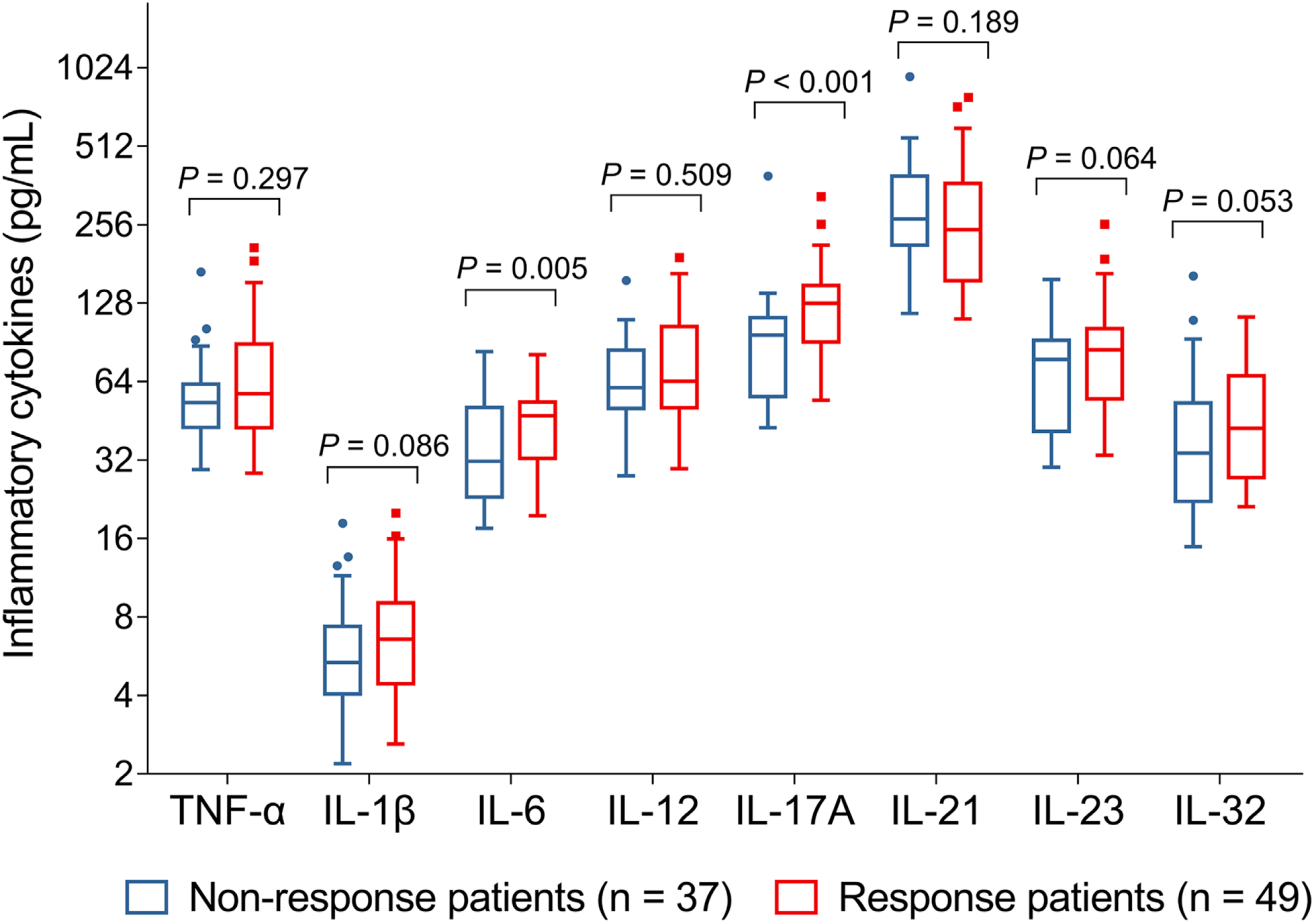
Comparation of baseline inflammatory cytokines levels between axSpA patients with and without ASAS40 response to adalimumab treatment at W12. ASAS40, Assessment in SpondyloArthritis International Society 40; axSpA, axial spondyloarthritis; IL, interleukin; TNF‐α, tumor necrosis factor α; W12, week 12

Baseline levels of IL‐6 (*P* = 0.005) and IL‐17A (*P* < 0.001) were positively correlated with ASAS40 response, whereas baseline levels of TNF‐α (*P* = 0.297), IL‐1β (*P* = 0.086), IL‐12 (*P* = 0.509), IL‐21 (*P* = 0.189), IL‐23 (*P* = 0.064), and IL‐32 (*P* = 0.053) were not associated with ASAS40 response in axSpA patients (Figure 2). The baseline IL‐6 and IL‐17 levels were higher in the responding axSpA patients compared with HCs (both *P* < 0.01, Figure S2).

### Independent predictive factors for ASAS40 response in axSpA patients

As listed in Table 2, univariate logistic regression analysis showed that baseline IL‐17A (odds ratio [OR] 1.014, *P* = 0.016) and IL‐6 (OR 1.043, *P* = 0.010) were correlated with a higher probability to achieve ASAS40 response in axSpA patients. However, other baseline inflammatory cytokines, including TNF‐α, IL‐1β, IL‐12, IL‐21, IL‐23, and IL‐32, were not associated with ASAS40 response in axSpA patients (all *P* > 0.05). As to other variables in the univariate logistic regression model, history of TNF inhibitor (OR 0.286, *P* = 0.014) was correlated with lower possibility to achieve an ASAS40 response in axSpA patients, meanwhile, baseline CRP (OR 1.047, *P* = 0.008) and ASDAS‐CRP (OR 2.184, *P* = 0.006) were correlated with higher probability to achieve ASAS40 response in axSpA patients.

**TABLE 2:** Logistic regression analysis for ASAS40 response at W12

Items	*P* value	OR	95% CI	
			Lower	Upper
Univariate logistic regression analysis				
Age	0.216	0.969	0.921	1.019
Male	0.151	2.428	0.723	8.155
Disease duration	0.384	0.954	0.859	1.060
History of TNF inhibitor	**0.014**	0.286	0.105	0.780
HLA‐B27‐positive	0.144	2.968	0.690	12.764
CRP	**0.008**	1.047	1.012	1.084
ESR	0.079	1.020	0.998	1.042
BASDAI score	0.177	1.387	0.863	2.228
BASFI score	0.126	1.390	0.912	2.119
Total back pain score	0.379	1.149	0.843	1.567
PGADA score	0.654	1.071	0.793	1.447
ASDAS‐CRP	**0.006**	2.184	1.245	3.831
TNF‐α	0.148	1.011	0.996	1.025
IL‐1β	0.170	1.091	0.963	1.235
IL‐6	**0.010**	1.043	1.010	1.077
IL‐12	0.263	1.008	0.994	1.021
IL‐17A	**0.016**	1.014	1.002	1.025
IL‐21	0.343	0.999	0.996	1.001
IL‐23	0.094	1.011	0.998	1.023
IL‐32	0.266	1.009	0.993	1.026
Forward stepwise multivariate logistic regression analysis				
History of TNF inhibitor	**0.029**	0.279	0.088	0.880
CRP	**0.012**	1.042	1.009	1.076
IL‐17A	**0.037**	1.013	1.001	1.026

Abbreviations: ASAS40, Assessment of Spondyloarthritis International Society 40; ASDAS‐CRP, Ankylosing Spondylitis Disease Activity Score with C‐reactive protein; BASDAI, Bath Ankylosing Spondylitis Disease Activity Index; BASFI, Bath Ankylosing Spondylitis Functional Index; CI, confidence interval; CRP, C‐reactive protein; ESR, erythrocyte sedimentation rate; HLA‐B27, human leukocyte antigen B27; IL, interleukin; OR, odds ratio; PGADA, Patient Global Assessment of Disease Activity; TNF, tumor necrosis factor.

To further evaluate the independent predictive factors for ASAS40 response, a forward stepwise multivariate logistic regression analysis was conducted, which disclosed that baseline IL‐17A (OR 1.013, *P* = 0.037) was independently associated with a higher possibility to achieve ASAS40 response in axSpA patients. As to other variables in the multivariate logistic regression model, the history of TNF inhibitor (OR 0.279, *P* = 0.029) was an independent factor in predicting a lower likelihood of achieving ASAS40 response in axSpA patients. In contrast, baseline CRP (OR 1.042, *P* = 0.012) was independently associated with a higher possibility to achieve ASAS40 response in axSpA patients (Table 2).

**FIGURE 3:**
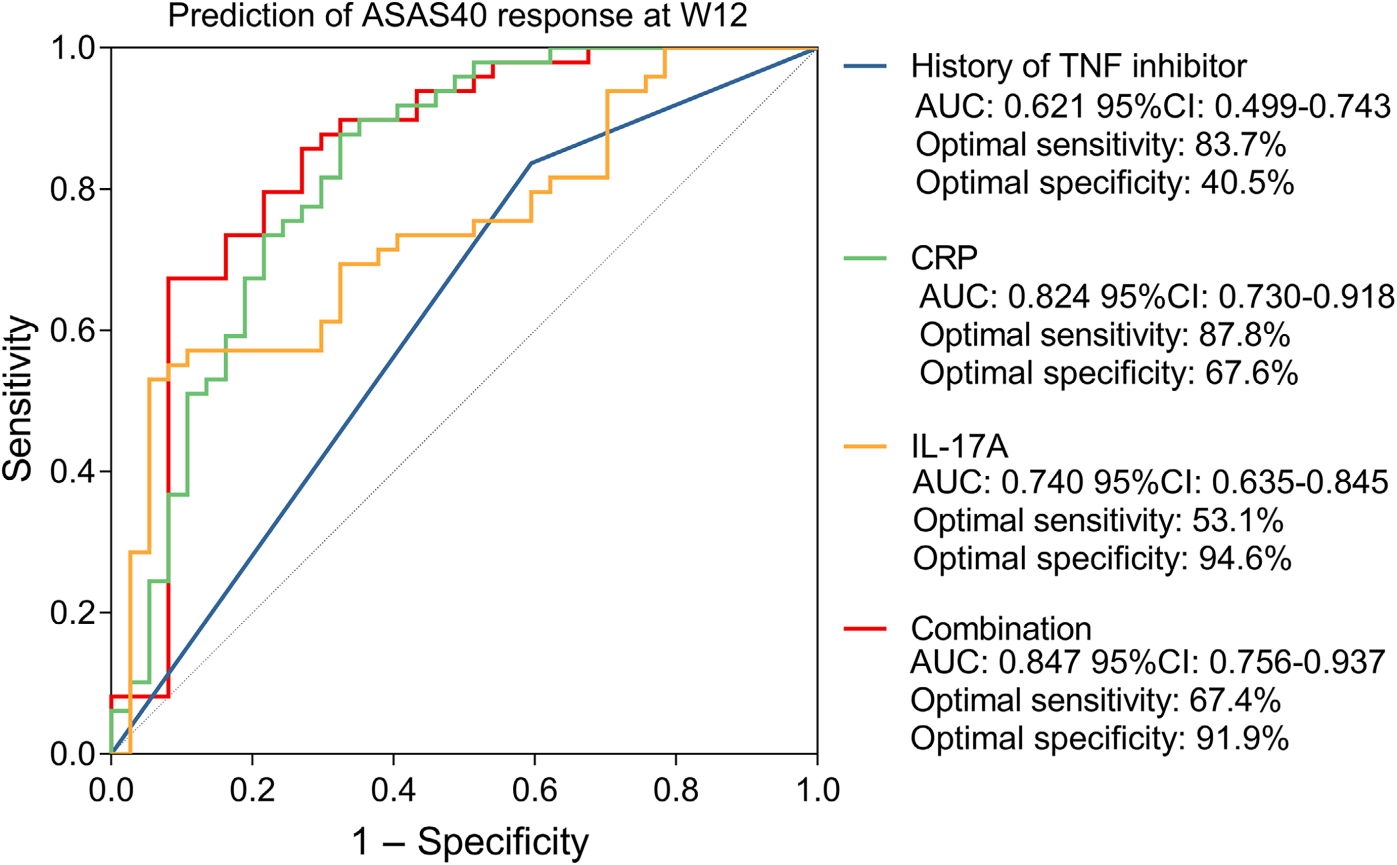
ROC curve analysis of history of TNF inhibitor, baseline CRP, baseline IL‐17A, and their combination. ASAS40, Assessment in SpondyloArthritis International Society 40; axSpA, axial spondyloarthritis; CRP, C‐reactive protein; IL‐17A, interleukin‐17A; ROC, receiver operating characteristic; TNF, tumor necrosis factor; W12, week 12

The ROC curve analysis showed that baseline IL‐17A, history of TNF inhibitor, baseline CRP, and their combination had an acceptable ability for predicting ASAS40 response of axSpA patients with areas under the curve of 0.740, 0.621, 0.824, and 0.847, respectively (Figure 3). Besides, this study also determined the ability of baseline IL‐6 for the prediction of ASAS40 response, which showed that its area under the curve was only 0.675 (Figure S3), which was numerically lower than the baseline IL‐17A.

## 4 DISCUSSION

Our study disclosed that more than 50% of axSpA patients achieved ASAS40 response at week 12 after adalimumab treatment. This discovery is similar to previous reports revealing that approximately 44.5%‐53% of patients realized ASAS40 response after 12 weeks of adalimumab treatment.^17^, ^18^ Furthermore, this finding may explain that adalimumab restrains the production of inflammatory cytokines by targeting TNF‐α, thereby further suppressing inflammation, reducing disease activity, and improving spine function in axSpA patients. Hence, axSpA patients respond well to adalimumab.

As for the correlation between the baseline levels of inflammatory cytokines and ASAS40 response, a recent study showed that after treatment with TNF inhibitors, axSpA patients with good response at week 12 have a lower baseline IL‐6 level. In contrast, the non‐response patients have a higher baseline IL‐6 level.^19^ In our study, the high baseline levels of IL‐6 and IL‐17A were correlated with ASAS40 response to TNF inhibitors in axSpA patients, whereas only baseline IL‐17 was independently associated with ASAS40 response to TNF inhibitors. The presumed interpretations are the following. (a) Patients with axSpA with higher baseline levels of IL‐17A have a more severe inflammatory condition compared with those with an attenuated level; meanwhile, TNF inhibitors may have a better anti‐inflammatory effect in the axSpA patients with a more severe inflammatory condition; thereby, axSpA patients with elevated baseline levels of IL‐17A achieve a higher ASAS40 response rate to TNF inhibitors.^20^ (b) The axSpA patients with higher baseline levels of IL‐17A may be more sensitive to TNF inhibitors, and so achieve a higher ASAS40 response rate after treatment with TNF inhibitors; however, this hypothesis needs further validation. (c) Baseline IL‐6 is not an independent factor predicting the ASAS40 response to TNF inhibitors for possible reasons: the correlation between baseline IL‐6 with ASAS40 response to TNF inhibitors might be weakened by other factors (such as the history of TNF inhibitor, baseline CRP, and baseline IL‐17A), so that in the multivariate model, baseline IL‐6 is not independently associated with the ASAS40 response.

Previous studies have evaluated the value of cytokine levels in predicting the response of axSpA patients to TNF inhibitors.^19^, ^21^, ^22^, ^23^, ^24^ For instance, one study showed that CRP and Jun N‐terminal kinase pathway‐associated phosphatase (JKAP) might predict the treatment response to TNF inhibitors.^21^ Another study exhibited that circulating baseline IL‐6 and erythrocyte sedimentation rate might reflect the response to adalimumab in AS patients.^19^ Partially in line with previous studies, our study found that high baseline levels of IL‐17A and CRP were independently correlated with a higher ASAS40 response rate. In comparison, the history of TNF inhibitor independently predicted a lower ASAS40 response rate. Besides, the ROC curve analysis showed that baseline IL‐17A, history of TNF inhibitor, baseline CRP, and their combination had acceptable excellent ability in predicting ASAS40 response. Their combination showed the most substantial ability in distinguishing patients with ASAS40 response from those without ASAS40 response. These results could be explained by the following. (a) IL‐17A plays a vital role in the development of inflammation in axSpA patients; meanwhile, a high baseline IL‐17A level indicates a more serious inflammatory condition, which intensifies the efficacy of the TNF inhibitors, thereby causing a higher ASAS40 response rate.^20^, ^25^ (b) CRP is a well‐known acute phase inflammatory marker, extensive studies disclose that its high baseline expression is related to an excellent response to TNF inhibitors in autoimmune diseases including RA and Crohn disease and axSpA.^21^, ^26^, ^27^ In axSpA patients, we suppose that a high baseline CRP level is correlated with aggravating disease status, including high disease activity and exacerbating inflammatory conditions; in addition, patients with disturbing disease status may benefit more from the TNF inhibitor treatment, thereby, leading to a higher ASAS40 response rate. (c) Patients with axSpA with a history of TNF inhibitor use may have a higher level of anti‐drug antibody compared with the TNF inhibitor naive patients, which would decrease the blood concentration of adalimumab, thereby causing a worse efficacy in these patients.^26^, ^28^, ^29^ (d) the combination of the history of TNF inhibitor, baseline CRP, and baseline IL‐17A is a good predictor for treatment response to adalimumab, which helps to identify the axSpA patients who would benefit from TNF inhibitor treatment, thereby, guiding the administration of personalized medicine. Therefore, the determination of these three indices (baseline IL‐17A, history of TNF inhibitor, and baseline CRP) might be more suitable for clinicians to determine whether the patient is going to respond to TNF inhibitor. However, more studies are needed to verify this finding.

There were some limitations of this study. The sample size was relatively small, which might cause a low statistical power. The axSpA patients enrolled in this study were young; therefore, the results of this study were not suitable for older axSpA patients. The ASAS40 response was evaluated after short‐term treatment (12 weeks), and the value of inflammatory cytokine levels for predicting long‐term treatment response to TNF inhibitor as well as the disease flare is not clear, which needed further evaluation. The inflammatory cytokine levels were only evaluated at baseline, but not during the follow‐up period, so the value of inflammatory cytokine levels in monitoring the progression of axSpA needs further exploration.

Measurement of pre‐treatment inflammatory cytokine levels is valuable for predicting treatment efficacy of TNF inhibitors in axSpA patients.

## AUTHOR CONTRIBUTIONS

YT designed the study; FP, FC, and YT performed the study and analyzed the data; FP, HW, and JB drafted the paper. All authors read and approved the paper.

## CONFLICT OF INTEREST

All authors declare no conflicts of interest.

## ETHICAL APPROVAL

This study was approved by Institutional Review Board, and each participant signed an informed consent.

## Supporting information


Figure S1

Figure S2

Figure S3
