# Supplementary figures and images for: Measurement of pre‐treatment inflammatory cytokine levels is valuable for prediction of treatment efficacy to tumor necrosis factor inhibitor in axial spondyloarthritis patients
Source: Int J Rheum Dis. 2022 Jun 12;25(8):844–50. doi: 10.1111/1756-185X.14353 (PMC9542033; doi:10.1111/1756-185X.14353)

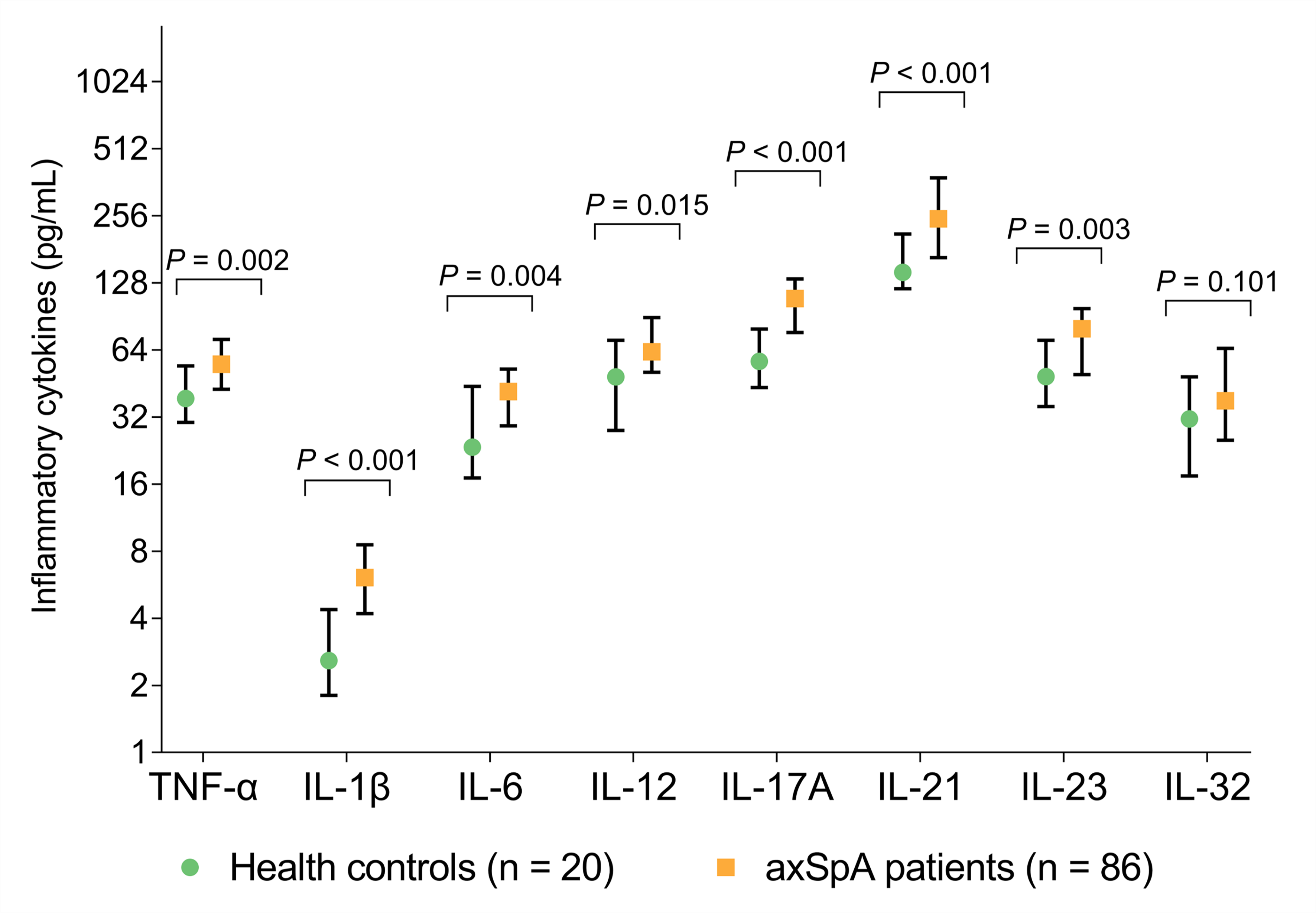

Supplement: Supplementary file 1 — Figure S1 [file APL-25-844-s002.tif]

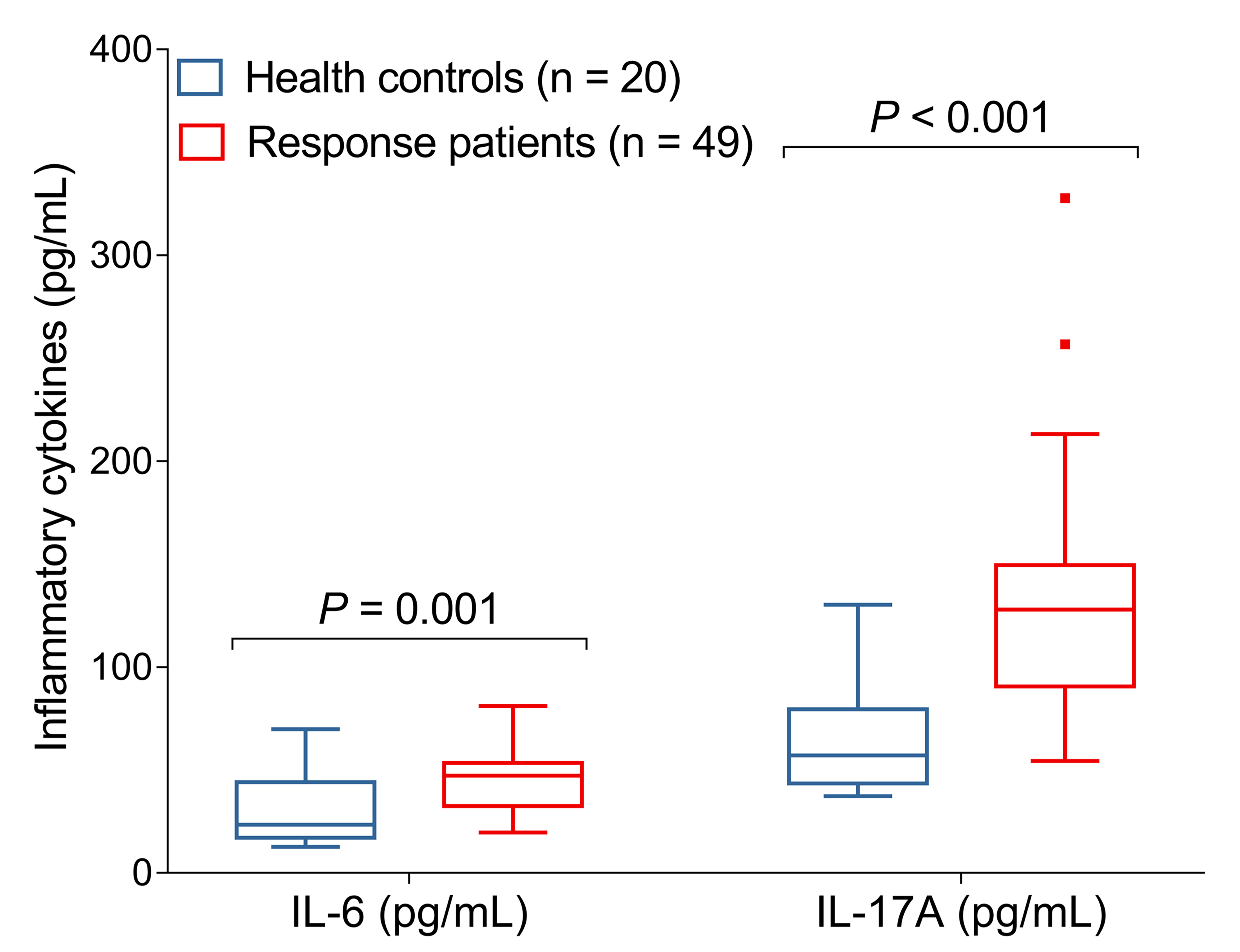

Supplement: Supplementary file 2 — Figure S2 [file APL-25-844-s003.tif]

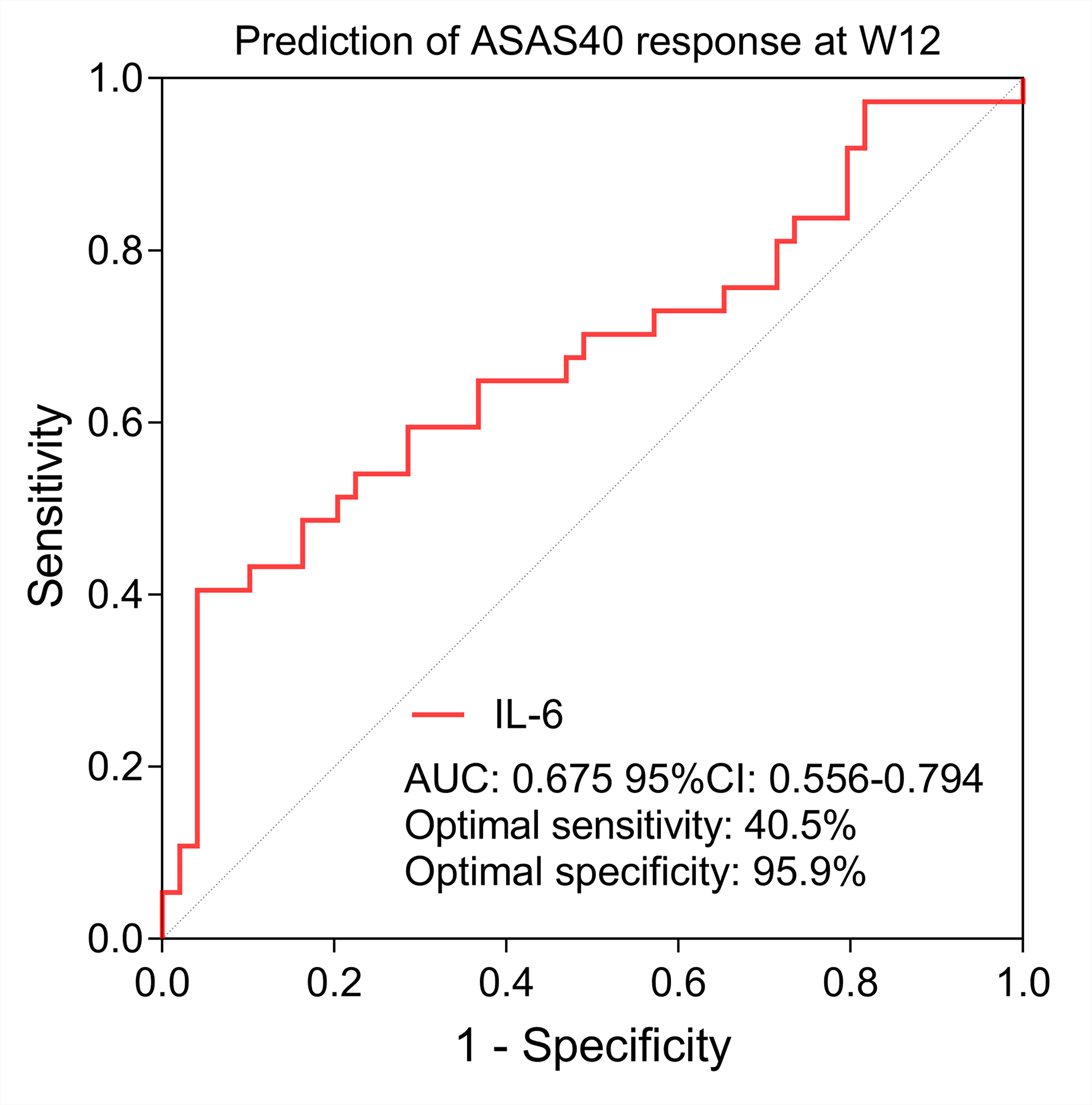

Supplement: Supplementary file 3 — Figure S3 [file APL-25-844-s001.tif]
